# Delay in antiretroviral therapy initiation is associated with worse mood symptoms in adults with chronic HIV disease

**DOI:** 10.64898/2026.10.07.26364968

**Published:** 2026-10-08

**Authors:** Hyunchan Lee, Claire O’Connor, Grace Hanly, Linxi Chen, Danial Mahmood, David J. Moore, Ronald J. Ellis, Xiong Jiang

## Abstract

**Background:**

In the era of successful antiretroviral therapy (ART), mood disturbance remains highly prevalent in people with HIV (PWH). In the present study, we investigated the long-term legacy effects of long delay in ART initiation and low nadir CD4 count on mood symptoms in adults with HIV. The impacts of demographic and other HIV clinical factors were also examined.

**Methods:**

A total of 109 PWH (disease duration: 24.6±9.3 years) and 42 demographically matched healthy participants (people without HIV, or PWoH) participated in a cross-sectional study (41-70 years old (55.6±6.8), 62.9% African Americans, 27.2% female, 14.3±2.9 years of education). Mood symptoms were assessed with the Beck Depression Inventory-II (BDI-II) and the Profile of Mood States (POMS). A comprehensive neuropsychological test battery was administered to assess neurocognitive function.

**Results:**

Despite largely comparable neurocognitive performance between PWH and PWoH, PWH had worse mood symptoms than PWoH, including higher total BDI-II ( *p* <.001) and total POMS scores ( *p=* .002), as well as higher score in all POMS sub-scores ( *p* <.007), except Vigor ( *p* =.724). Within the PWH group, longer delays in ART initiation, lower nadir CD4 count, and lower educational attainment were associated with worse mood symptoms. By contrast, current plasma CD4+ cell count, plasma detectable viral load, and HIV disease duration were not associated with either BDI-II or POMS score.

**Conclusion:**

Mood disturbance is highly prevalent in PWH on ART. The long-term legacy effects of delay in ART initiation and low nadir CD4 count on mood management reinforce the necessity of early ART initiation in clinical practice.

## Full Text Availability

The license terms selected by the author(s) for this preprint version do not permit archiving in PMC. The full text is available from the preprint server.

